# A Rare Case of Verrucous Carcinoma and Trichoblastoma of the Neck: A Case Report and Literature Review

**DOI:** 10.7759/cureus.38236

**Published:** 2023-04-28

**Authors:** Yesenia Brito, Samira Zubair, Zaid Taki El-Din, Janice Mwaniki, Ameya Nair, Frederick Tiesenga

**Affiliations:** 1 Surgery, St. George's University School of Medicine, True Blue, GRD; 2 Surgery, Saint James School of Medicine, Arnos Vale, VCT; 3 General Surgery, West Suburban Medical Center, Chicago, USA

**Keywords:** hybrid verrucous tumors, unusual presentation, composite tumor, neck tumors, cutaneous squamous cell carcinoma, trichoblastoma, verrucous carcinoma

## Abstract

Verrucous carcinoma (VC) is a variant of cutaneous squamous cell carcinoma. This phenomenon mainly affects the oropharynx, genitalia, and soles of the feet. VC is a well-defined, exophytic, cauliflower-like growth that is warty in nature. Trichoblastoma is a benign epithelial tumor composed of follicular germinative cells. It presents as a small, smooth, non-ulcerated, skin-colored nodule on the scalp, neck, thigh, and perianal regions. The dual presentation of verrucous carcinoma and trichoblastoma of the neck is rare. Though treatment can be achieved through surgical resection, early detection offers a good prognosis.

We present the case of a 54-year-old homeless male who presented with an unusual neck mass that was initially misidentified as an abscess. Surgical debridement was performed, and histopathological analysis revealed the presence of a rare combination of VC and trichoblastoma. This report highlights the challenges of this rare presentation, which may be overlooked or misdiagnosed as an abscess.

## Introduction

This case report presents the rare finding of verrucous carcinoma (VC) and trichoblastoma in a neck abscess. Verrucous carcinoma is a subtype of squamous cell carcinoma, usually found in the oral cavity [1]. It is characterized by a slow-growing, locally invasive tumor that is typically not aggressive and rarely metastasizes. Trichoblastoma is a rare benign tumor that arises from the hair follicles [2].

Our patient presented with a neck abscess that was found to be malignant upon histological examination. The presence of both verrucous carcinoma and trichoblastoma malignancy in abscesses is uncommon [1,2]. The identification of verrucous carcinoma was established by the detection of well-differentiated squamous cells that showed minimal atypia and keratinized cells. The diagnosis of trichoblastoma was made based on the presence of atypical cells with features of trichoblastic differentiation.

Identifying these malignancies in a neck abscess highlights the importance of conducting thorough histological examinations in cases of chronic abscesses. Additionally, it emphasizes the necessity for further research to gain a better understanding of the correlation between neck abscesses and the emergence of malignancies. Early detection and treatment are crucial in preventing malignancy progression and improving patient outcomes.

## Case presentation

**Figure 1:**
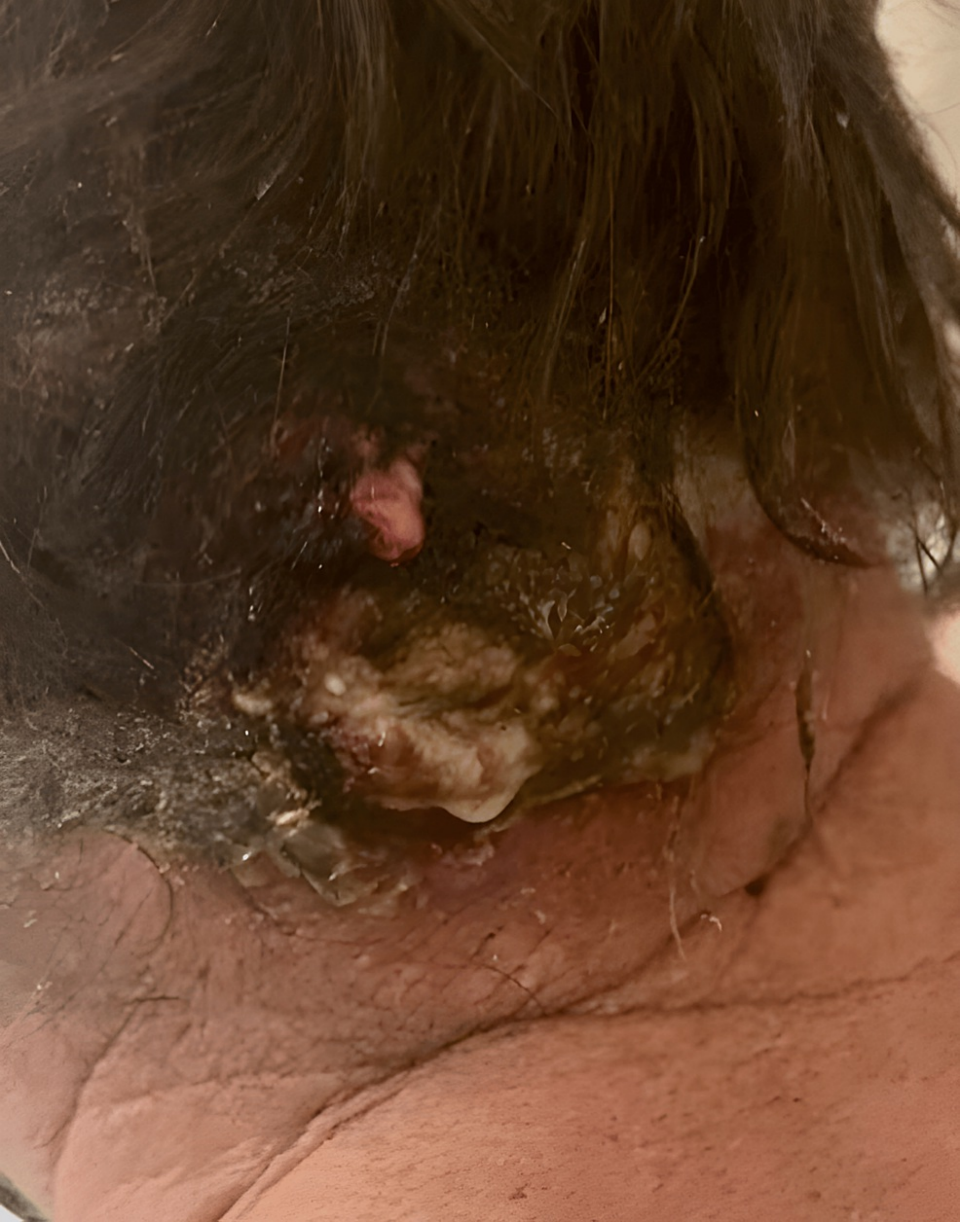
Irregular outgrowth lesion with a yellowish green purulent material

A 54-year-old homeless male presented to the emergency department (ED) complaining of a painful and purulent abscess on the left occipital scalp and posterior neck (Figure 1). The pain was localized around the lesion, with a yellow purulent discharge expressed with gentle pressure. The patient noticed the “bump” a week prior to his ED visit. He denied fever, chills, headache, or neck pain. The patient’s social history was significant for one pack per day, current everyday smoker, and daily alcohol consumption. No relevant past medical history was reported.

**Figure 2:**
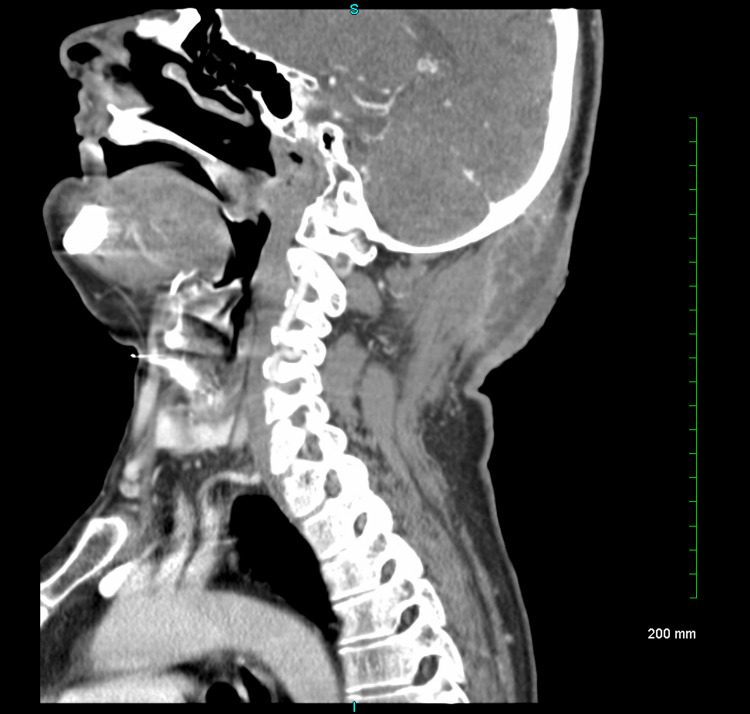
Soft tissue abscess identified posteriorly measuring up to 5.5 cm, which extends in proximity to the occipital bone on the left with no definite erosion to suggest osteomyelitis

On admission, a computed tomography (CT) scan of the neck with contrast showed a soft tissue abscess in proximity to the occipital bone (Figure 2).

**Figure 3:**
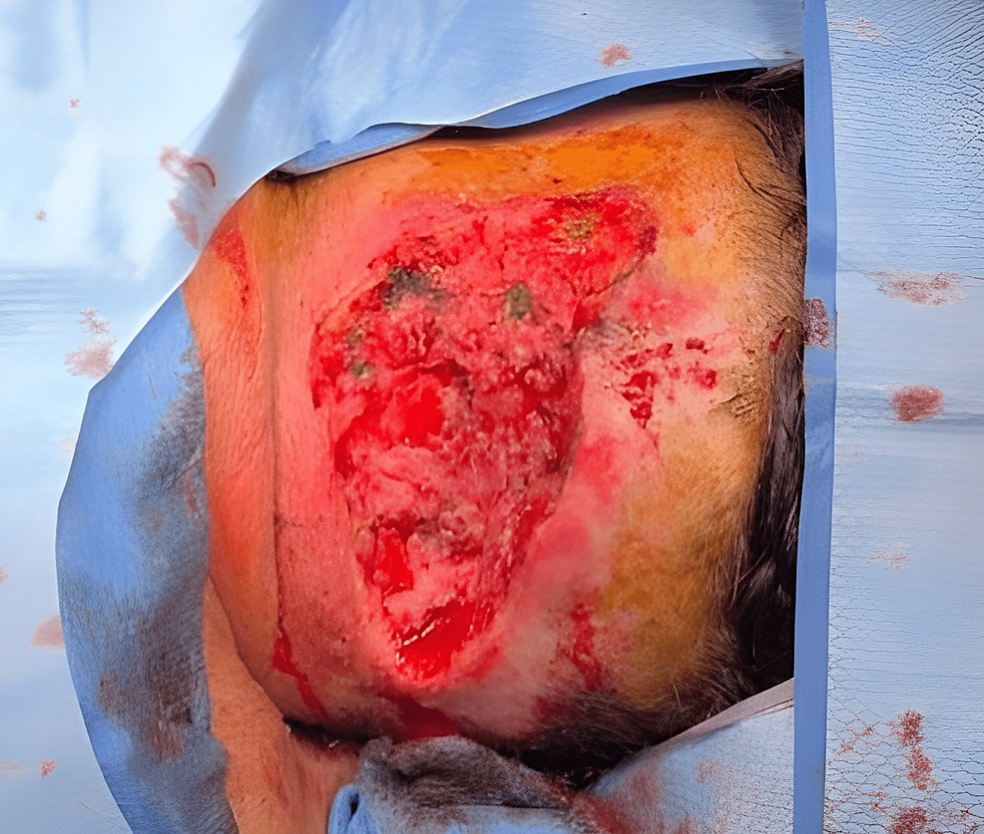
Incision and debridement, 10 x15 cm in dimension, of the left occipital and upper neck abscess

The patient was taken to the operating room (OR) the day following his admission. An extensive and liberal excision of the diseased skin and subcutaneous tissue with adequate margins was undertaken (Figure 3). All infected tissue was resected and sent for histological evaluation. A large amount of purulent discharge was encountered and cultures were taken. Hemostasis was assured with cautery and the wound was packed open.

Wound cultures from ED encounters as well as cultures from surgical debridement grew methicillin-resistant Staphylococcus aureus (MRSA). Ceftriaxone and vancomycin were switched to doxycycline 100 mg twice a day as indicated by infectious disease.

**Figure 4:**
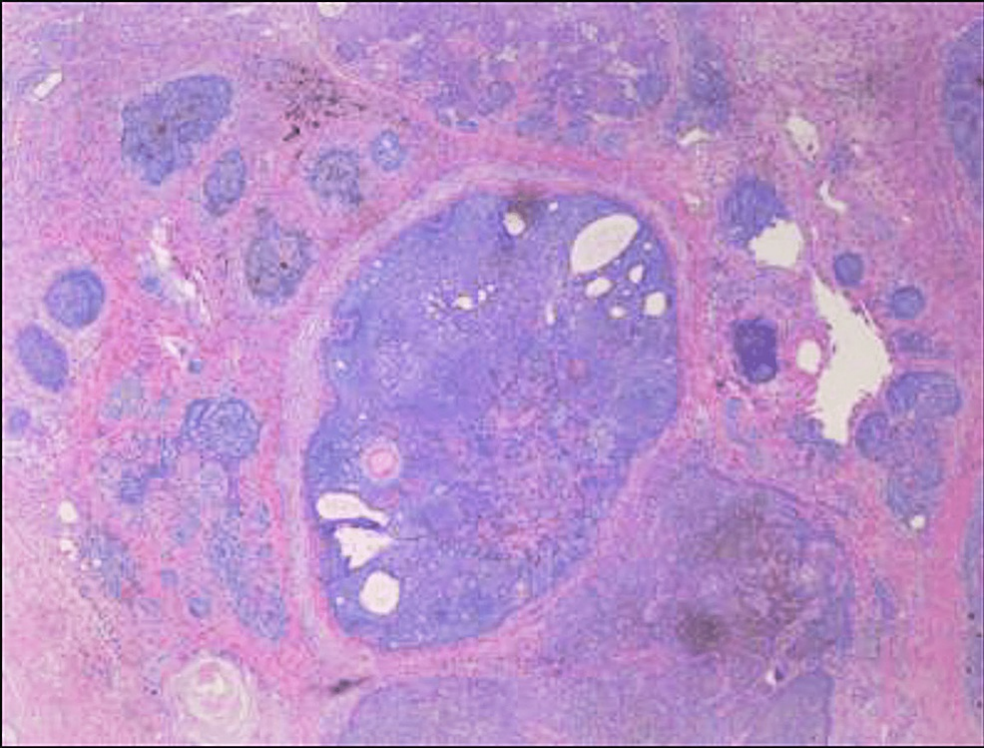
Verrucous carcinoma and trichoblastoma/trichoepithelioma within a background of nevus sebaceous The resection margin is free of the tumor.

**Figure 5:**
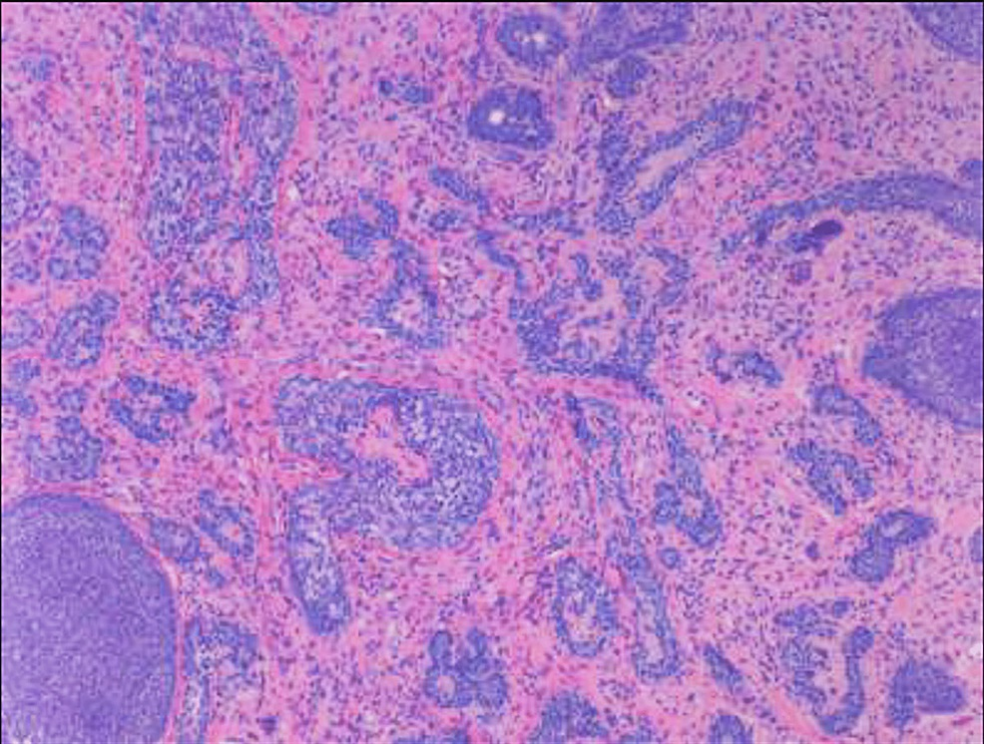
Predominant (skin with subcutaneous) tissue with abscess, severe acute and chronic inflammation, and reactive change

Histopathology report showed verrucous carcinoma and trichoblastoma/trichoepithelioma with background nevus (Figures 4, 5). A hematologist-oncologist was consulted.

On postoperative day (POD) four, wound vacuum-assisted closure (VAC) was recommended and placed on the incision site; however, it was discontinued on the same day, as the patient could not tolerate the machine due to pain. The transition to daily dressings change was started.

Incision examination on POD seven showed a necrotic slough wound, for which wound re-debridement was performed. The patient’s postoperative course was uneventful. The patient was discharged three days after his last debridement. Discharge instructions were given to the patient, and he was advised to establish care in Cook County Hospital with primary care providers. A shelter referral was offered, however, the patient declined and requested resources for the bus.

## Discussion

The uniqueness of this case highlights the incidence of verrucous carcinoma and trichoblastoma occurring together on a sebaceous nevus at the nape of the neck region. Both are subtypes of squamous cell carcinomas that, when combined, are referred to as “hybrid” verrucous carcinomas [3]. These hybrid cases, making up 20% of verrucous carcinomas, are typically classified as squamous cell carcinomas, but a case could be made for a distinctive classification [4]. On presentation, it was broad-based with a brown fungating appearance. It also had a yellow-green pustular exudate with focal hemorrhage. Histopathology classified it as a verrucous carcinoma and trichoblastoma on a background sebaceous nevus. Additionally, the patient reports that the carcinoma occurred rapidly over the course of a week. Typically, the mean duration of the lesion at the time of diagnosis is 13 to 16 years [5].

VC presents as a broad-based, cauliflower-like benign tumor with sharply defined margins [6]. It is characterized by a slow growth pattern that is markedly invasive into cartilage and bone but has minimal metastatic potential [6]. Proximal lymph node hyperplasia may occur in reaction to mononuclear inflammation along the tumor border [6].

VC is most common in the oral cavity and larynx and is highly associated with tobacco use, poor oral hygiene, and HPV [7]. Despite this, it only makes up 2-12% of all oral carcinomas [8]. In less than 10% of cases, it may appear on the ear, lip, face, sole of the foot, bladder, and genitals [3,9]. It is identified as more prevalent in males in comparison to females (60%), with a mean age of 69 [6].

Alternatively, trichoblastoma originates from follicular germ cells [10]. It is classified as a benign subtype of squamous cell carcinoma. It infiltrates the dermis and subcutis and primarily localizes to the head and neck [10]. Various cases of trichoblastoma and other cutaneous neoplasms have been reported [10]. There is a high correlation with the formation of trichoblastoma on a background sebaceous nevus, but there are also occurrences in isolation. A histopathological diagnosis is necessary due to its clinical similarity to basal cell carcinoma [11].

The treatment of choice for both verrucous and hybrid verrucous carcinomas is wide surgical excision, excluding lymph node resection due to no nodal involvement [4]. Radiotherapy is debated as a treatment choice, as some research points to the possibility of anaplastic transformation as well as the high risk of failure, complications, and decreased survivability [3,6,12]. Irrelative to treatment choice, a high local recurrence ranges from 30-50%, possibly due to inadequate surgical resection and the size of the tumor [3].

The hybrid verrucous carcinoma was further complicated by MRSA infection, requiring treatment with antibiotics. While verrucous carcinomas have a favorable prognosis, their deeply penetrating character and proximity to the patient's occipital bone made them a good choice for excision and debridement. As clinical and pathological data are needed to accurately diagnose and resect the hybrid verrucous tumors, careful examination is necessary.

The importance of suspecting a malignancy in bizarre presentations cannot be overrated. Being vigilant for the possibility of malignancy in an unusual presentation is crucial for early detection and timely treatment of skin cancers. This may involve performing a biopsy and getting histopathology on board to confirm the diagnosis, which can improve the prognosis and potentially save the patient's life.

## Conclusions

Verrucous carcinoma is a type of squamous cell carcinoma that typically targets the oral cavity; however, in our patient, it is identified in the back of the neck. Multiple risk factors like alcohol consumption, poor hygiene, and smoking predisposed our patient to the development of verrucous carcinoma. Currently, there is no established medication-based treatment plan for malignancy presenting as an abscess. The sole course of action is often surgical excision and radiation therapy of affected areas. If the disease progresses to metastatic disease or penetrates into unresectable areas, this could lead to a poor prognosis for the patient. Further studies are required to determine valid and rational treatment approaches and mitigate the likelihood of metastatic malignancy development.
